# Workplace Mental Health Interventions in India: A Rapid Systematic Scoping Review

**DOI:** 10.3389/fpubh.2022.800880

**Published:** 2022-05-03

**Authors:** Apurvakumar Pandya, Niharika Khanal, Mudita Upadhyaya

**Affiliations:** ^1^Parul Institute of Public Health, Faculty of Medicine, Parul University, Vadodara, India; ^2^Public Health Researcher, Ghaziabad, India; ^3^Public Health Researcher, Germantown, TN, United States

**Keywords:** workplace mental health, employee wellbeing, India, work place and improvement, wellbeing cost

## Abstract

The mental health initiatives at the workplace are growing in numbers over the past few years. Public and private sectors continue to explore avenues to navigate and adapt initiatives to promote employee's mental wellbeing. However, such initiatives in the Indian context are not thoroughly studied. We attempted to review existing literature on workplace mental health interventions in the Indian context. The scoping review was conducted following the standard process as recommended by the Joanna Briggs Institute and the Preferred Reporting Items for Systematic Review and Meta-Analysis extension for scoping reviews. We searched in the databases such as PubMed, Google Scholar and Scopus. Scientific literature including gray literature of the past decade was searched to synthesize evidence on types of mental health interventions and their unique features. Of the 1,311 records, 30 records that met the inclusion criteria were included for the final review. The review highlights evidence on stress and mental health problems faced by the working population and various strategies adopted by organizations to address mental health problems. However, very few interventions were accompanied by comprehensive needs assessment, impact evaluation and workplace policy initiatives. Most interventions were curative–provisioning counseling services, limiting the scope of mental health promotion activities. Addressing mental health wellbeing comprehensively and aligning an organization's policies are crucial. Research on employee mental health, its risk factors, and cost-effectiveness analysis of workplace mental health interventions in the Indian context need to be prioritized.

## Introduction

Mental health problems in the working population (15–64 years of age) are growing public health concerns. Recent evidence indicates that non-communicable diseases (NCDs) including mental health problems such as stress, depression, and anxiety among working populations are directly linked to reduced work performance and increased absenteeism (1). Studies also show that investment in such interventions is cost-saving (2).

Mental disorders in the working population are a major cause of disability and unemployment (3, 4). The World Health Organization (WHO) estimates that the burden of mental health problems in India generates as high as 2,443 disability-adjusted life years (DALYs) per 100,000 population followed by the estimated economic loss, between 2012 and 2030, at USD 1.03 trillion (5). Importantly, most NCDs including mental disorders occur in people below 60 years of age, in other words, among the working-age population (6). The global cost of mental health conditions in 2010 was estimated at US$ 2.5 trillion, with the cost projected to surge to US$ 6.0 trillion by 2030, which is a huge burden on society (7). Thus, a multipronged approach to address NCDs including mental health problems among the working population commands urgent attention.

### Workplace Mental Health in Indian Context

According to Census 2011 (8), India has a 474 million working population. Furthermore, the country's Sample Registration System's 2018 report (9) shows that country's demographic dividend continues to grow. The proportion of the working-age population is expected to increase from 61% in 2011 to 65% in 2036, adding 12 million people to the working population each year.

Mental disorders are impacting millions of working populations in India. Although there are no population-based prevalence studies for working populations, there are estimates of mental disorders in India, implying the burden of mental disorders in the working population. For example, the findings of the National Mental Health Survey 2015-16 (10) by the National Institute of Mental Health and Neuro-Sciences (NIMHANS) estimated that nearly 150 million individuals suffer from one or the other mental disorders of varying severity, comprising 10.5% of the population. In 2017, the Global Burden of Disease Study published in 2020 (6) revealed 197 million people experiencing mental illnesses, comprising 14.3% of the total population. Both reports implicitly imply that the majority of the population with mental disorders in the working-age groups, between 15 and 59 years of age.

The survey by The7th Fold 2020 (11) with 509 working people across metros cities and diverse sectors from India revealed that 36% of employees were suffering from one or other types of mental health issues. The situation of mental health has been exacerbated due to the COVID-19 pandemic, making it a more serious concern. Recent PwC's 2021 Employee Financial Wellness Survey (12) reported that 63% of employees were experiencing stress due to financial strain ever since the COVID-19 pandemic began. Another study (13) conducted during the second wave of the pandemic by Deloitte ranked India as the highest among 18 countries in terms of anxiety. These studies suggest that workplace mental health requires immediate attention.

Poor mental health at the workplace can be a contributor to a range of physical illnesses like hypertension, diabetes and cardiovascular conditions, amongst others (14). Recent evidence indicates that employee effectiveness resonates with the mental health of the employees, and it contributes to overall organizational productivity (15). Thus, prioritizing the mental health of employees is essential.

Much of the literature on workplace health and mental health promotion interventions originate from high-income countries with a long history of industrial development. These countries have made workplace health a priority and expanded government as well as private health-insurance regulations, which perhaps contributed to the growing number of workplace health interventions (16–21). Despite the extensive global literature on workplace mental health initiatives and improvement in employee's productivity, the extent of workplace mental health interventions in India remains unknown. Further, evidence on the effectiveness of workplace interventions particularly in India is scarce. Therefore, the present rapid systematic scoping review attempts to review existing evidence on workplace mental health intervention in India and identify unique features.

## Methods

### Study Design

This rapid systematic scoping review aims to identify and summarize diverse evidence on workplace mental health promotion programs in India and their unique features. This rapid review followed the standard process as recommended by the Joanna Briggs Institute (22) and adhered to the Preferred Reporting Items for Systematic Review and Meta-Analysis (PRISMA) extension for scoping reviews (PRISMA-ScR) (23).

### Definition of Population, Intervention Comparator and Outcomes (PICO) of the Study

- Population: Employees–primarily working population – (15–64 years of age) in India.
- Intervention: Any interventions introduced by the organization to address mental health problems such as stress, psychological distress or promote mental wellbeing in the Indian organizational set-up.
- Comparators: Not applicable for this scoping review.
- Outcome: Types of mental health interventions, key features of mental health interventions, best practices and effect on mental health status if reported.

### Search Strategy

A systematic search of the literature was conducted across four databases: PubMed, Scopus, Google Scholar, and PsycINFO. Keywords such as “employee wellbeing program,” “employee wellness,” “workplace mental health interventions,” and “India” were used and searches were narrowed by using Boolean operators. Two researchers conducted a literature search and the third researcher reviewed the screening, resolved conflicts related to the final selection of records. Searches were limited to articles published until October 20, 2021.

### Inclusion and Exclusion Criteria

All records were imported in Microsoft excel and duplicates were removed. Unqualified records were excluded based on the exclusion criteria.

Inclusion criteria of the study:

1. Studies conducted with the working population in India.
2. Any studies (quantitative, qualitative, or mixed-method studies) were included.
3. Studies explaining or describing employee wellbeing programs targeting mental health, key characteristics of different employee wellbeing programs and their effectiveness.
4. Studies published in English.
5. Peer-reviewed articles, gray literature such as project reports, annual reports explaining mental health interventions or employee assistance programs, and journal blogs.

Exclusion criteria of the study:

1. Publications published in other than the English language.
2. Publications such as perspective, editorial, and commentary.
3. Studies conducted in other country contexts and/or conducted with non-Indian participants.

### Screening of Records

We piloted 10 records against a priori inclusion and exclusion criteria to minimize selection bias. Each record title was reviewed by two independent screeners (AkP, NK). A third reviewer (MU) reviewed conflicts and resolved disagreements through discussion. Two reviewers also independently screened the full text of potentially eligible articles to check whether the records fulfilled the inclusion criteria.

### Data Extraction

We used Microsoft Excel to organize and code data used in the scoping review. Selected records were extracted in the domains such as author's name, year of publication, type of publication, country of the study conducted, type of population, mental health problems, different types of mental health interventions, key findings, and recommendations.

### Quality Assessment of Studies

The quality of included quantitative studies was assessed using the Effective Public Health Practice Project quality assessment tool (24). The studies are evaluated in eight parameters such as study design, analysis, withdrawals and dropouts, data collection practices, selection bias, invention integrity, blinding as part of a controlled trial, and confounders. Evaluation of each parameter range between “strong,” “moderate,” and “weak.” Qualitative studies were assessed using the Critical Appraisal Skills Programme quality assessment tool (25). The studies are evaluated based on 10 questions followed by reviewer's overall comments based on responses to 10 questions.

### Data Synthesis

Full text of selected records was appraised and thematically organized. Both authors reviewed the data synthesis. Thematic analysis methods (26) were used to identify key themes across studies. Thematic analysis was used as most of the studies were descriptive in nature. A mind map was constructed to sort the type of features of workplace mental health interventions and their unique features. Overarching themes were then reviewed, refined, and named (27). Results were synthesized in five themes namely: classification of mental health interventions, quality assessment of studies, evidence on mental health problems of the working population, workplace mental health interventions, and outcomes of mental health interventions.

## Results

**Figure 1:**
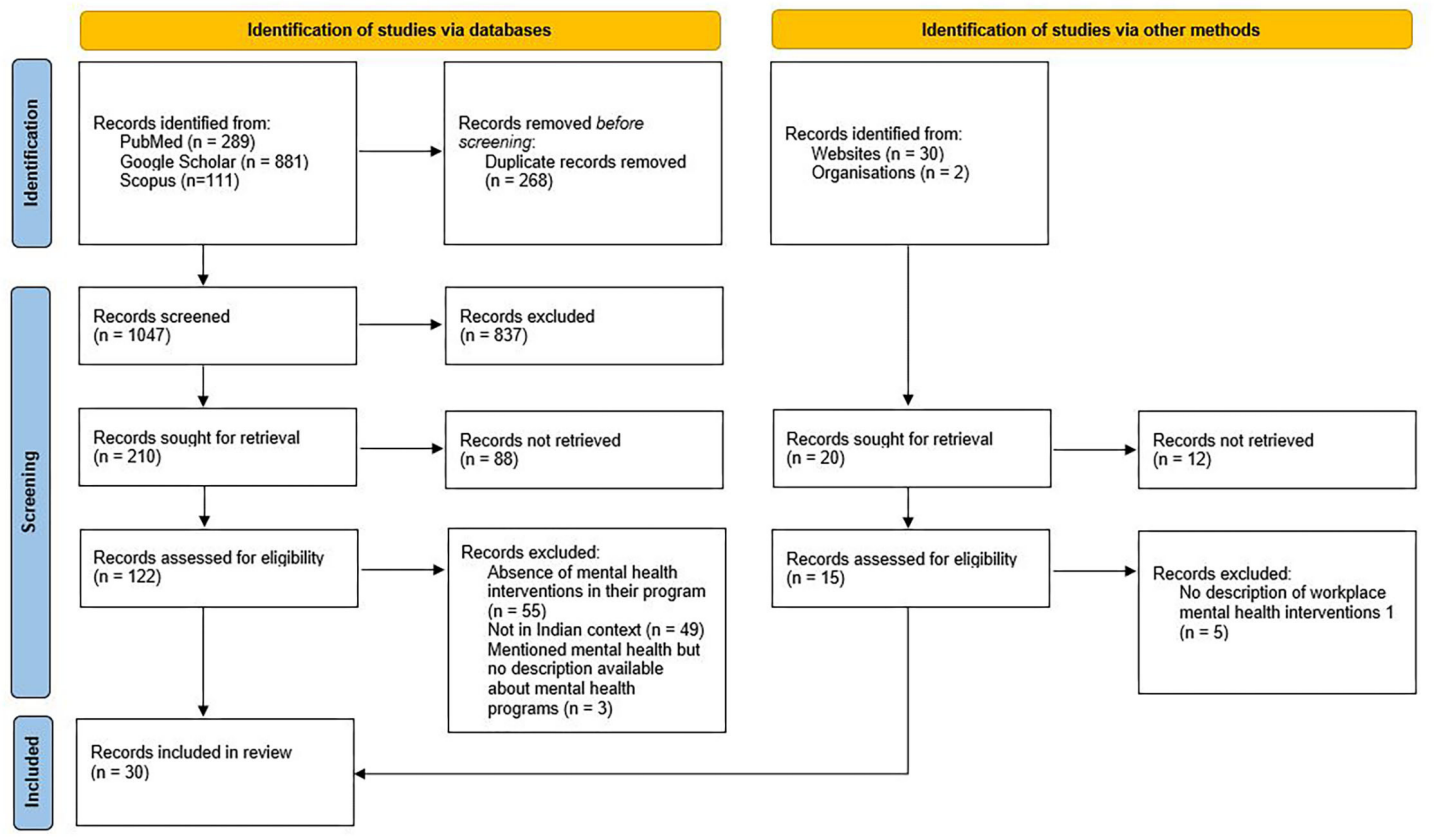
PRISMA flow diagram of the study.

The literature search resulted in 1,311 records. After removing duplicates, 1,047 were screened for the title. Of the 137 records screened for full-text review, 30 records met inclusion criteria and were finally included for the review. Figure 1 provides a summary of the PRISMA flow diagram.

Mental health interventions were further classified as (1) corporate wellness programs, (2) employee assistance programs and (3) health promotion interventions. Table 1 presents the type of mental health interventions and corresponding numbers of studies.

**Table 1:** Classification of workplace mental health interventions.

**Sr. No**.	**Type of mental health interventions**	**Key features**	**No. of records**
1	Corporate wellness program	• Work-life balance strategies (such as wellbeing related leaves, ‘no meeting Friday's policy, etc.) • Wellness sessions (yoga/meditation/stress management sessions etc.)	15
2	Employee assistance program	• Counseling services outsourced • Some offer counseling services through Mobile application, 24*7 tele helpline	8
3	Employee wellbeing program	• Physical healthcare services • Mental health services	3
4	Peer Support program	• Mental health advocates • Peer support • Peer-to-peer counseling	4

### Quality Assessment of Studies

Out of 30 records, 7 were observational studies, 5 were reviews, 3 qualitative studies and 15 were websites and e-newspaper articles. We found that the overall methodological quality of observational studies was moderate to weak. About 5 papers were rated at moderate whereas, 2 were rated as weak studies. None of the studies were rated to be of strong. The Critical Appraisal Skills Programme quality assessment tool evaluated three qualitative studies to be average.

Anecdotal evidence of the ability to make positive impacts on improving employee's mental health outcomes and overall wellbeing were noted in websites and e-newspapers; however, a lack of methodological rigor was a common problem. Overall, there was a lack of properly controlled studies to attribute the mental health outcomes or employee wellbeing to the interventions only.

### Evidence on Stress and Mental Health Problems of the Working Population

Occupational stress or work-related stress is a significant cause of poor physical and mental health, low productivity, and human error. The World Health Organization defines occupational stress as “…the response people may have when presented with work demands and pressures that challenge their ability to cope” (3, 28). Occupational stress emerged as the most common risk factor contributing to increased sickness absence, high staff turnover, sub-optimal performance and a possible increase in accidents due to human err (4, 5).

Factors such as workload, lack of participation and control in the workplace, monotonous tasks, role ambiguity or role conflict, lack of recognition at work, poor interpersonal relationships, poor working conditions, poor communication, and conflicting home and work demands contribute to stress (16–19). Bullying and sexual harassment at the workplace are also critical causal factors of chronic stress (3). It is evident that chronic stress leads to a range of physical illnesses like hypertension, diabetes, cardiovascular conditions as well as mental health problems such as burnout, psychological distress, anxiety or depression, significantly affecting employees ability to contribute meaningfully in their personal and professional lives (16, 18, 19). Worryingly, these health problems especially in the working population is on the rise (28).

Employees with mental illnesses also face discrimination at the workplace. Brouwers et al. (29) have conducted a cross-sectional study that reported that about 67% of employees who suffered from depression either experienced discrimination at the present workplace or faced discrimination while applying for new jobs. The stigma and discrimination push the working population to suffer silently and avoid seeking professional mental health help (30). A study conducted by ASSOCHAM in 2015 (31) found that 43% of employees in the private sector experienced signs of general anxiety disorder or depression. A survey conducted last year (11, 32) in India revealed that more than a quarter of Indian employees (36%) were experiencing mental health problems, and half of the sample (50%) were worried about an uncertain future due to the COVID-19 pandemic (32). Certainly, the negative impact of the COVID-19 outbreak on mental wellbeing is undeniable.

### Workplace Mental Health Interventions in India

Indian multinational companies have been prominent in using the workplace to promote long-term health behavioral change to the measurable benefit of themselves, their employees and local communities. Supplementary Table 1 highlights notable employee wellbeing programs in India (33–49).

As shown in Supplementary Table 1, many companies (*n* = 15) offer preventive and promotive physical and mental healthcare services, few companies (*n* = 4) emphasize work-life balance in addition to counseling services. Some companies (*n* = 5) have strengthened primary mental healthcare through training employees on psychological first-aid and establishing peer-support networks within the company. One company has upgraded employee health insurance and provisioned reimbursement for mental health consultations and another offering counseling services through an Artificial Intelligence (AI) based chatbot (2). Many companies (*n* = 7) use the digital platform to provide mental healthcare services Many other companies do implement workplace mental health interventions but at a smaller scale. For example, the companies such as ICICI Lombard, Capegemini India, Oyo, Uber India, Google India, Mondelez India, American Express India and Panasonic India offer mental health counseling by outsourcing services under employee assistance programs (33). Many consulting agencies such as BetterLYF (34), MeeHappy (35), Trueworth wellness (36), YourDost (37), WYSA (38), ePsyClinic (38), Trijog (38), Mindhouse (39), Mind Care India (40), Practo (41) and Optum (42) provide mental health services for the national and multi-national companie's staff.

### Outcomes of Workplace Mental Health Interventions

Although outcomes of these programs in the Indian context are not yet published, such programs potentially prevent the breakdown of the employees. The global literature highlights that employee wellbeing programs (which include mental health interventions) have prevented employees from mental illnesses (43–49). Moreover, employee wellbeing programs (with a focus on mental health promotion) have been proved to be cost-saving; companies save anywhere between $3 to 15 per for every 1$ spent on workplace mental health programs (50). The regular physical activities and health check-ups were found promising in early diagnosis, treatment and controlling NCDs (43).

Employee wellbeing programs also improve employee engagement significantly (51, 52). Employee recognition and constructive feedback practices too contribute to higher employee engagement levels (51). Most importantly, employee wellbeing programs improve productivity and morale. When employee wellbeing is optimized, employees focus more on their work, and their productivity increases (49). The available evidence highlights that health promotion activities, psychosocial intervention along stress management training have a positive impact on mental wellbeing (53–55).

A recent survey report of human resources leaders (48) from 400 organizations across 15 different industries in India revealed half of the organizations offer health benefits as screenings or health awareness programs to employees, dedicated resources to address the spectrum of wellbeing, including mental wellbeing. However, impact assessment, documentation of workplace mental health interventions was lacking, only 40% of organizations have a documented wellbeing plan (53).

## Discussion

Organizations in India have certainly realized the need for protecting employee's mental health and have started offering policies and programs that promotes positive mental health. Most programs are more employee growth oriented and family-friendly. Although, numerous organizations have introduced policies to ensure the physical wellbeing of their employees, very few organizations have explicit policies for mental health promotion and programs addressing employees' mental wellbeing. Those organizations having employee wellbeing programs, ironically, revealed difficulties in engaging their employees in employee wellbeing programs (11, 33, 54–64). Perhaps, mental health stigma precludes employees identify and seeks timely mental health help. Efforts are needed to create mental health literacy at workplaces. Moreover, a proactive approach to mental health at the workplace targeted to create safe and conducive environment is commanded.

While research has established the link between wellbeing (which encompasses positive mental health) and productivity, organizations need to enforce guidelines based on acceptable industrial practices for maintaining an optimal work-life balance.

Any wellbeing initiatives that are designed with the employee's needs in mind can certainly thrive. Thus, assessing employees need and their participation in designing and implementing mental health interventions is crucial. Larger companies may benefit from “decentralizing and allowing regional business units to design and customize the execution of mental health interventions” can potentially increase employee participation rates.

Psychological capital (such as efficacy, hope, optimism, and resilience) (65, 66) can be fostered through mental health promotion interventions such as imparting life skills, stress management, fostering work-life balance, encouraging physical activities and create conducive environment through peer support. Strengthening psychological capital has direct association with positive attitude, productivity and mental wellbeing (66).

### Implications for Policy, Practice, and Research

Employee wellbeing programs can potentially impact employee's experience. Many strategies organizations can implement to improve their employee's wellbeing.

Organizations need to introduce policy-level changes to address employee wellbeing. Without clearly articulated workplace policy, employee wellbeing initiatives may not be sustainable and impactful. The development and execution of a workplace mental health policy and employee wellbeing program will benefit the health of employees, increase the productivity of the organization (2, 3).

Employees need may vary depending up on their occupations and job roles. Thus, comprehensive needs assessment is recommended prior designing and implementing workplace mental health program. Some additional strategies for fostering positive mental health are enumerated as below:

- Encourage collaboration in the organization to boost teamwork, competitiveness, and employee morale. By enabling easy collaboration, organizations can leverage employee's diverse skill sets, feel recognized, and decrease their stress levels.
- Formulate grievance redressal mechanism to listen and address employee's concerns. Employees want to be listened to.
- Promote regular and constructive feedback. Regular feedback provides scope for improvement, advancing their skills and make them feel satisfied with their jobs. The feeling of satisfaction and learning attitude is crucial for employee wellbeing.
- Train employees on psychological first-aid and peer counseling. There is a tremendous need to make workplaces more humane. Training employees on psychological first-aid and peer counseling destigmatize mental health and empower them to seek timely support at the primary level. It enables timely referral for treatment when needed. Moreover, this will equip employees to show empathy and compassion for others, ultimately creating an emotional bond between employees and employers, and making workplace a better place to work.
- Introduce periodic general health screening. Ensuring annual health screening can help identify diseases early and treat employee's health issues timely.
- Establish in-house psychological counseling services or formulate linkages with trained mental health service providers. By offering psychological counseling services, organizations can reduce stigma.
- Other strategies such as flexible work options, introducing health initiatives like an organization-wide competition in running or cycling, and promoting mental wellbeing mobile applications can help cope with stress and improve quality of life.
- Promote research. Organizations can establish linkages with academic and research institutions to carry out researches including impact assessment periodically.

### Strengths and Limitations of This Study

A rapid systematic scoping review was conducted to map workplace mental health interventions, identify key features of these interventions and gaps in workplace mental health interventions. In order to ensure the relevance of our review, the review scope, review questions, protocol and literature search strategies were defined based on the targeted literature review conducted by both authors. The study provides a descriptive synthesis of current evidence on interventions to prevent mental health for working people. Most details regarding mental health interventions found from gray literature i.e., e-magazines and websites, left us with limited information about the interventions. The authors did write to the company for detailed information; however, only two companies responded to our queries. Further, few studies used cross-sectional surveys, making it difficult to determine the longitudinal impact. Despite these limitations, the present study provides a mapping of workplace mental health interventions in Indian companies and offer insights on key features and presented strategies to address gaps in workplace mental health interventions.

## Conclusions

A healthy population productively contributes to the economy and it is in the benefit of organizations to safeguard public health. Given the substantial contributions of the private sector to the economy, making an investment in formal and structured workplace mental health interventions should be a strategic priority. There is a need to map the impact of workplace mental health programs on productivity and health outcomes. Also, documenting best practices around workplace mental health interventions and positive case stories are worthy exercises. Research on employee mental health, associated risk factors, and cost-effectiveness analysis should be promoted.

## Data Availability Statement

The original contributions presented in the study are included in the article/Supplementary Material, further inquiries can be directed to the corresponding author.

## Author Contributions

AkP conceptualized, prepared the protocol, and revised the manuscript. NK and AkP carried out the literature search and screened records for inclusion in the study. MU synthesized the evidence and drafted the manuscript. All authors have reviewed, edited, and approved the submitted version of the manuscript.

## Conflict of Interest

The authors declare that the research was conducted in the absence of any commercial or financial relationships that could be construed as a potential conflict of interest.

## Publisher's Note

All claims expressed in this article are solely those of the authors and do not necessarily represent those of their affiliated organizations, or those of the publisher, the editors and the reviewers. Any product that may be evaluated in this article, or claim that may be made by its manufacturer, is not guaranteed or endorsed by the publisher.
