# Supplementary material for: Workplace Mental Health Interventions in India: A Rapid Systematic Scoping Review
Source: Front Public Health. 2022 May 3;10:800880. doi: 10.3389/fpubh.2022.800880 (PMC9110774; doi:10.3389/fpubh.2022.800880)
Supplement: Supplementary file 1 [file Table_1.docx]

### Supplementary Table 1. Workplace mental health interventions in Indian organizations

| **Sr. No.** | **Organizations** | **Brief description of Intervention** | **Best Practices** | **Reference** |
| --- | --- | --- | --- | --- |
| 1 | Apollo Life | Offers three healthcare services:   1. Health services such as various blood tests, scans, Ayurveda and beauty packages 2. Nutrition advisory services (such as health or dietary advice) 3. Mental health services in a form of personalized counseling sessions | - Integration of Ayurveda for healthcare services - Tele-counseling services - De-addiction programs | 33 |
| 2 | Genpact | 1. Senior leaders communicate often through various channels across all levels 2. Does regular virtual connects 3. Virtual counseling services via AI chatbot (Amber) to check in with employees to capture immediate concerns and needs to support them better. | - Preventive and promotive mental healthcare - Regular communication with staff - AI chatbot for counseling services | 33 |
| 3 | Wipro | 1. Full health check-ups and coverage for the basic treatments and surgeries at discounted price 2. Mitr (Friend) initiative to provide counseling for employees of the company | - Peer support system to offer basic counseling services through peers | 33 |
| 4 | Larsen & Toubro Infotech (L&T) | Healthcare programs considering long term illnesses.Integrates seamlessly technology with healthcare to provide optimal benefits to employees  1. In-house employee counseling | - Digital interface for accessing healthcare services from their empanelled healthcare service providers - Employee counseling | 33 |
| 5 | Mphasis | 1. Developed its wellness app  A telephone support line with professional counsellors | - Dedicated mental health program - Use of technology for mental wellbeing | 37 |
| **Sr. No.** | **Organizations** | **Brief description of Intervention** | **Best Practices** | **Reference** |
| 6 | Agilent Technologies | 1. Fosters employee work-life balance by providing freedom and flexibility in deciding time and place of work to its employees 2. Rewards employee performance rather than working a greater number of hours | - Work-life balance - Employee reward based on performance | 37 |
| 7 | Zensar Technologies | 1. Provides crèche facility to employees 2. Arranges informal meetings where employees can meet their seniors and discuss anything and everything 3. Provides information services such as information regarding housing/education facilities for children 4. Organizes stress management programs 5. Arranges social events such as family day and fun days at work regularly 6. A facility called “Madat Online” – a 24/7 service available for employees to take care of some of their personal day-to-day activities (e.g., dropping cheques, drawing cash, paying telephone bills and school fees of employees’ children) | - Employee friendly support services such as childcare service, personal day-to-day support service and so | 37 |
| 8 | Shell | 1. Practices employee diversity and work-life balance; employees are given options such as flextime and work from home. 2. Initiatives such as live meditation, extended weekends, special holidays, free subscription to wellbeing apps 3. Mental health counseling sessions | - Employee diversity - Telephone helpline (24x7) - Use of digital platforms - wellbeing apps | 39 |
| **Sr. No.** | **Organizations** | **Brief description of Intervention** | **Best Practices** | **Reference** |
| 9 | IBM India | 1. Flexible workweek schedules, working from home, part-time employment, and leave of absence programs and so 2. Family counseling 3. Other provisions include study reimbursements, global opportunities, recreational activities 4. Help employees deal with life events, from getting married to taking a career break | - Provide a range of practical supports and preventive services - Counseling services extended to family members - Flexi schedules for employees | 39 |
| 10 | Edelweiss Group | 1. Regular employee outreach programs 2. Townhalls by the leadership 3. Virtual counseling | - Regular communication with staff - Virtual counseling | 40 |
| 11 | MakeMyTrip | 1. Organise emotional counseling sessions 2. Yoga and meditation sessions by experts 3. Stress and anxiety management consultations by expert | - Emotional wellness sessions - Stress and anxiety management services | 40 |
| 12 | Snapdeal | 1. Provide access to licensed counsellors 2. Organize meditation and breathwork classes | - Mental health services by mental health professionals | 40 |
| 13 | Adobe India | 1. Day offs every third Friday 2. Free access to meditation and wellbeing applications like Headspace and LifeDojo etc. | - Digital healthy lifestyle promotion | 40 |
| 14 | Publicis Sapient | 1. Mental health first aiders 2. Regular sessions with specific focus on women, people facing domestic violence, etc. | - Peer support services through Mental health first-aiders - Regular sessions on sensitive topics and addresses vulnerability of women at workplace | 40 |
| **Sr. No.** | **Organizations** | **Brief description of Intervention** | **Best Practices** | **Reference** |
| 15 | Diageo | 1. Employee assistance program to provide counseling services 2. Conduct mental wellness sessions for subordinates covering topics such as effective stress management, avoiding digital burn-out among other issues while for line managers, sessions focus on team management and promoting mental wellness across the team | - Mental wellness sessions for subordinates as well as line managers | 27 |
| 16 | Tata Consultancy Services (TCS) | TCS offers primary following services:   1. Regular health screening and counseling services 2. Regular sessions of physical activities (yoga & meditation) 3. Stress management and sleep management sessions 4. Deliver well-being nudges digitally to employees 5. ‘Maitree’ is an initiative for the spouses of employees 6. Trained managers as “Emotional Health first aiders” to provide psychological first aid to colleagues 7. Involve employees in social responsibilities like community development programs | - Regular counseling services - Physical activities sessions - Stress management and sleep management sessions promote positive health at workplace - Peer support system in the form of creating a pool of emotional health first-aiders - Use of technology to promote positive mental health - The domain of welfare practices extended to outside the workplace by engaging employees in social responsibilities | 38 |
| 17 | HCL Technologies | 1. Counseling services for employees 2. Coaching services | - Dedicated mental health program   Offers coaching for personal and professional development   - Counseling through smartphone apps, offering sessions through chatbots or one-to-one counseling sessions | 39 |
| **Sr. No.** | **Organizations** | Brief description of Intervention | **Best Practices** | **Reference** |
| 18 | Infosys | 1. Health Assessment and Lifestyle Enrichment (HALE) initiative to foster employee health, quality of life, and work environment 2. HALE Hotline – 24*7 telephone counseling services 3. InfyTV shows through dedicated TV channel for promoting healthy lifestyles 4. Infy Radio shows featuring interviews with doctors, mental health experts and promotes HALE’s wellbeing activities among employees 5. Mobile app – InfyFit that includes health trackers, tools and wellness challenges 6. Desktop app -Blink O Wink App for the employee to remind digital detox, drink water, exercise etc. | - It has a holistic approach to promote a positive environment through collaborative care and a range of wellness management programs | 40 |
| 19 | Mahindra & Mahindra | “M Happy initiative” offers counseling services through Employee Assistance Program | - Employee counseling service | 42 |
| 20 | JSW Group | 1. We Care program offer counseling services through Employee Assistance Program 2. Telephone counseling services | - Employee counseling service | 42 |
| 21 | CSS Corp | 1. Round-the-clock employee assistance program that provides counseling services 2. The [HR chatbots automate responses](https://analyticsindiamag.com/chatbots-in-mental-health-friendly-but-not-too-friendly/) to questions, accelerating resolutions 3. Conducting virtual mindfulness sessions 4. Digital detox to enable work-life balance | - Use of technology to promote mental wellbeing - Addressing HR-related queries - Digital detox | 42 |
| **Sr. No.** | **Organizations** | **Brief description of Intervention** | **Best Practices** | **Reference** |
| 22 | InMobi | 1. Instructor-led yoga & meditation session 2. ‘No meeting Fridays’ policy 3. 21 additional days of well-being related leaves 4. Sessions by trained mental health professionals | - Employee well-being related leaves | 42 |
| 23 | Intuit | 1. Employee Assistance Program provides face-to-face counseling with qualified mental health experts 2. A digital mindfulness platform through the ‘Well-Mind’ program 3. Offer an annual reimbursement to cover wellbeing related expenses | - Counseling services by mental health experts - Use of digital platforms for promoting wellness | 42 |
| 24 | Accenture | Live Well program to provide tools, resources, and incentives for their employees to achieve physical, mental and financial health, as well as to achieve overall work-life balance and wellness goalsMental health advocatesUpgraded medical insurance policy by including mental health consultancy reimbursements for employees as well as dependent family membersProvisioning healthcare services through modern treatments as well as alternative treatments such as Ayurveda and homoeopathy | - Offers modern as well as alternative healthcare treatments - The Live Well program is for each employee, their spouse or domestic partner for a personal health assessment, wellness incentives and so - Inclusion of mental health consultancy reimbursements for employees as well as dependent family members - Peer support in terms of mental health advocates who regularly reach out to their peers | 42 |
| **Sr. No.** | **Organizations** | **Brief description of Intervention** | **Best Practices** | **Reference** |
| 25 | Thoughtworks | 1. Basic peer-to-peer counseling sessions 2. Care program to support professional and personal development and well-being 3. One-on-one personalised counseling sessions by trained mental health professionals 4. Focused sessions on wellness 5. Physical activity sessions | - Office initiatives for employees to promote openness at the workplace and discuss issues around mental health - Peer support services - Promotion of wellness and healthy lifestyles | 42 |
